# Derivation of the evolution of empathic other-regarding social emotions as compared to non-social self-regarding emotions

**DOI:** 10.1186/1471-2202-13-S1-P28

**Published:** 2012-07-16

**Authors:** Nicoladie D Tam

**Affiliations:** 1 Department of Biological Sciences, University of North Texas, Denton, TX 76203, USA

## 

The present study derives the evolution of social emotions by inclusion of other-regarding concerns from the non-social emotions of self-regarding concerns. Emotional processing is a self-discovered error-correction feedback process in which computations are involved to assess the accuracy of the internal brain-generated predictions with respect to the reality, in order to increase its probability of an organism’s own survival.

The current model extends the self-regarding emotional feedback for self-preservation to other-regarding emotional feedback for social-preservation by including “self-and-others” as an entity for survival optimization. It extends the previous emotion model, in which EMOTION-I [1] derived the pre-processing computations needed to assess the desirability of contextual “feel” of the sensory stimuli for survival. It further extends the EMOTION-II [2] model that derived the needed computations for self-discovery and self-correction of error-conditions in its internal predictions of the external world. This self-assessment is accomplished by comparing expected outcomes with actual outcomes, in which “disparity” represents a fault-condition leading to unhappiness as a feedback, and congruency leading to happiness as feedback for emotional processing [3].

The previous models focused on the self-preservation and self-survival as the criteria for error-minimization of the desirable outcome as predicted by the internal brain model for an autonomous organism. The present model incorporates the preservation of the social entity – i.e., the survival of the whole (between self and others), rather than the survival of the individual entity. By incorporating of the extended-self (that includes others) in the optimization process of error-minimization, the model provides the evolutionary process for social interaction that produces social emotions as feedback for the system (self and others) as a whole in its computation.

By extending the optimization process to include preservation of self and others, it introduces emotional feedback that is not just self-regarding, but also other-regarding. This forms the basis for social interactions that promote mutual survival, and the ability to “feel for others” – i.e., the development of empathy emotion.

This model is derived based on the ability for an organism to change the “frame of reference” from “self-centered” reference-frame to “other-centered” reference-frame. This change in reference-frame is computed by using the relativity principle of directional vectors that represent the disparity between self and others. When the frame of reference is switch from self to others, the directional vector could change sign from positive to negative, resulting in relativistic change in perception of the comparison between self and other – i.e., from self-centered, self-regarding optimization to other-centered, other-regarding optimization.

Furthermore, the objectivity of emotional response can be accomplished by using a neutral frame of reference that is neither self-centered nor other-centered, but with respect to an independent frame of reference from a neutral party. For instance, fairness is an assessment comparing the disparity between self and others, in which the disparity is also a signed vector quantity – unfairness is reflected by the negative disparity-vector, whereas fairness is reflected by the positive disparity-vector between self and others. The empathic emotion is an emotional feedback that can be computed by switching the frame of reference from self-centered to other-centered, such that perspective-switching can be viewed from the other’s point-of-view (putting in someone’s shoes), resulting in empathic emotional feedback. Thus, what is seen as unfair would become fair when such perspective (vectorial sign) is changed by the switch in reference-frame.

## References

[B1] Tam D EMOTION-I Model: A Biologically-Based Theoretical Framework for Deriving Emotional Context of Sensation in Autonomous Control Systems The Open Cybernetics & Systemics Journal 2007 1 28 46 10.2174/1874110X00701010028

[B2] Tam D EMOTION-II Model: A Theoretical Framework for Happy Emotion as a Self-Assessment Measure Indicating the Degree-of-Fit (Congruency) between the Expectancy in Subjective and Objective Realities in Autonomous Control Systems The Open Cybernetics & Systemics Journal 2007 1 47 60 10.2174/1874110X00701010047

[B3] Tam DN Computation in Emotional Processing: Quantitative Confirmation of Proportionality Hypothesis for Angry Unhappy Emotional Intensity to Perceived Loss Cognitive Computation 2011 3 2 394 415 10.1007/s12559-011-9095-2

